# Functional Interactions of *Kluyveromyces lactis* Telomerase Reverse Transcriptase with the Three-Way Junction and the Template Domains of Telomerase RNA

**DOI:** 10.3390/ijms231810757

**Published:** 2022-09-15

**Authors:** Wasif Al-Shareef, Yogev Brown, Christopher Bryan, Elena Shuvaeva, Shhadeh Bsoul, Raanan Greenman, Majdi M. Kabaha, Nikolai B. Ulyanov, Emmanuel Skordalakes, Yehuda Tzfati

**Affiliations:** 1 Department of Genetics, The Silberman Institute of Life Sciences, Edmond Safra Campus, The Hebrew University of Jerusalem, Jerusalem 91904, Israel; 2 Gene Expression and Regulation Program, The Wistar Institute, 3601 Spruce Street, Philadelphia, PA 19104, USA; 3 Department of Pharmaceutical Chemistry, University of California, San Francisco, CA 94158, USA

**Keywords:** yeast, ribonucleoprotein, telomerase, telomerase RNA, three-way junction, Est2, crystal structure

## Abstract

The ribonucleoprotein telomerase contains two essential components: telomerase RNA (TER) and telomerase reverse transcriptase (TERT, Est2 in yeast). A small portion of TER, termed the template, is copied by TERT onto the chromosome ends, thus compensating for sequence loss due to incomplete DNA replication and nuclease action. Although telomerase RNA is highly divergent in sequence and length across fungi and mammals, structural motifs essential for telomerase function are conserved. Here, we show that Est2 from the budding yeast *Kluyveromyces lactis* (*kl*Est2) binds specifically to an essential three-way junction (TWJ) structure in *K. lactis* TER, which shares a conserved structure and sequence features with the essential CR4-CR5 domain of vertebrate telomerase RNA. *kl*Est2 also binds specifically to the template domain, independently and mutually exclusive of its interaction with TWJ. Furthermore, we present the high-resolution structure of the *kl*Est2 telomerase RNA-binding domain (*kl*TRBD). Mutations introduced in vivo in *kl*TRBD based on the solved structure or in TWJ based on its predicted RNA structure caused severe telomere shortening. These results demonstrate the conservation and importance of these domains and the multiple protein–RNA interactions between Est2 and TER for telomerase function.

## 1. Introduction

**Figure 1:**
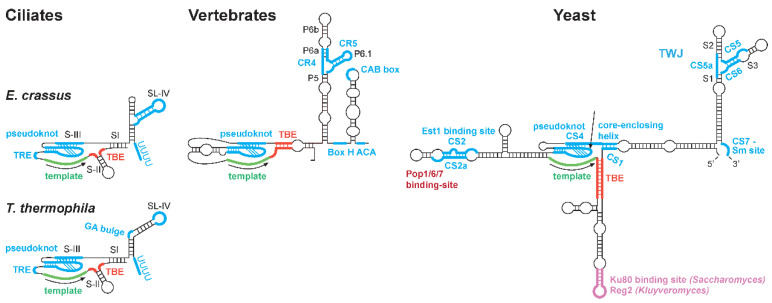
**Common secondary structure models for telomerase RNA.** Shown are predicted models for the ciliates *Tetrahymena thermophila* and *Euplotes crassus*, vertebrates and budding yeast TERs. Indicated are conserved regions and sequences (CR and CS), pairings/stems (P/S), stem-loops (SL), template recognition element (TRE), template boundary element (TBE) and three-way junction (TWJ).

Telomeres, the nucleoprotein structures that protect the ends of eukaryotic chromosomes, consist of short (5–26 nts), tandem, species-specific DNA repeats and associated proteins (reviewed in [1,2]). Telomerase is a ribonucleoprotein (RNP) reverse transcriptase that synthesizes these telomeric repeats onto the telomeric 3′ overhang to compensate for incomplete replication and exonuclease degradation (reviewed in [3,4]). Although significant progress has been made in understanding the structure and function of telomerase, its assembly, mechanism of action and regulation are not fully understood. In vitro telomerase activity using a telomeric oligonucleotide as a substrate depends only on the reverse transcriptase catalytic subunit (TERT; Est2 in yeast) and telomerase RNA (TER; TLC1 in *Saccharomyces cerevisiae*). However, in vivo elongation of the telomere requires additional interacting factors, such as the yeast proteins Est1, Est3, Cdc13 and the RNase P subunits Pop1, Pop6 and Pop7 [5,6,7]. In addition to providing the template for telomeric DNA synthesis, TER provides binding sites for telomerase proteins and regulators and facilitates the assembly of an active telomerase RNP complex [8]. Additional structure motifs, such as the triplex-containing pseudoknot and the template boundary element (TBE), regulate telomerase action along the template (Figure 1 and [9,10,11,12,13]).

During the assembly of the telomerase RNP, the *Tetrahymena* TERT interacts with the TER stem-loop IV and TBE [14,15], and the human TERT interacts with the CR4-CR5 domain and with the template-pseudoknot domain (Figure 1 and [16]). High-resolution structures have provided insights into telomerase complex assembly in the flour beetle *Tribolium castaneum,* the ciliate *Tetrahymena thermophile, Medaka* fish, human and, recently, also fungi [17,18,19,20,21,22,23,24,25,26,27]. In *S. cerevisiae*, regions important for the interaction of Est2 with TLC1 were identified [28,29], but the direct interaction sites and the overall structure of the assembled complex have not been characterized.

We previously identified, by phylogenetic analysis of six *Kluyveromyces* TERs, a three-way junction (TWJ) structure (Figure 1) and showed that it is essential for telomerase function [30]. Despite the high divergence in TER sequences, TWJ is conserved in structure and key residues in all other fungal TERs examined and also with the vertebrate CR4-CR5 domain (Figure 1 and [6,30,31,32]). Although *Tetrahymena thermophila* TER does not appear to contain a similar TWJ structure, TER sequences from other ciliates such as *Euplotes crassus* are predicted to form TWJ structures in the stem-loop IV domain (Figure 1). Here, we aimed to examine whether the structural conservation of these fungal, ciliate and vertebrate elements reflects a conserved function. We show that *K. lactis* Est2 (*kl*Est2) binds directly to *the K. lactis* TWJ (*kl*TWJ), similar to the binding of the vertebrate TERT with the CR4-CR5 domain [21,33,34,35]. In addition, *kl*Est2 also binds independently to the template domain. Altogether, our results indicate that telomerase RNA is more conserved in structure, protein–RNA interaction and function across vertebrates, yeast and even ciliates than previously appeared.

## 2. Results

### 2.1. The Precise TWJ Structure Is Important for In Vivo Telomerase Function

**Figure 2:**
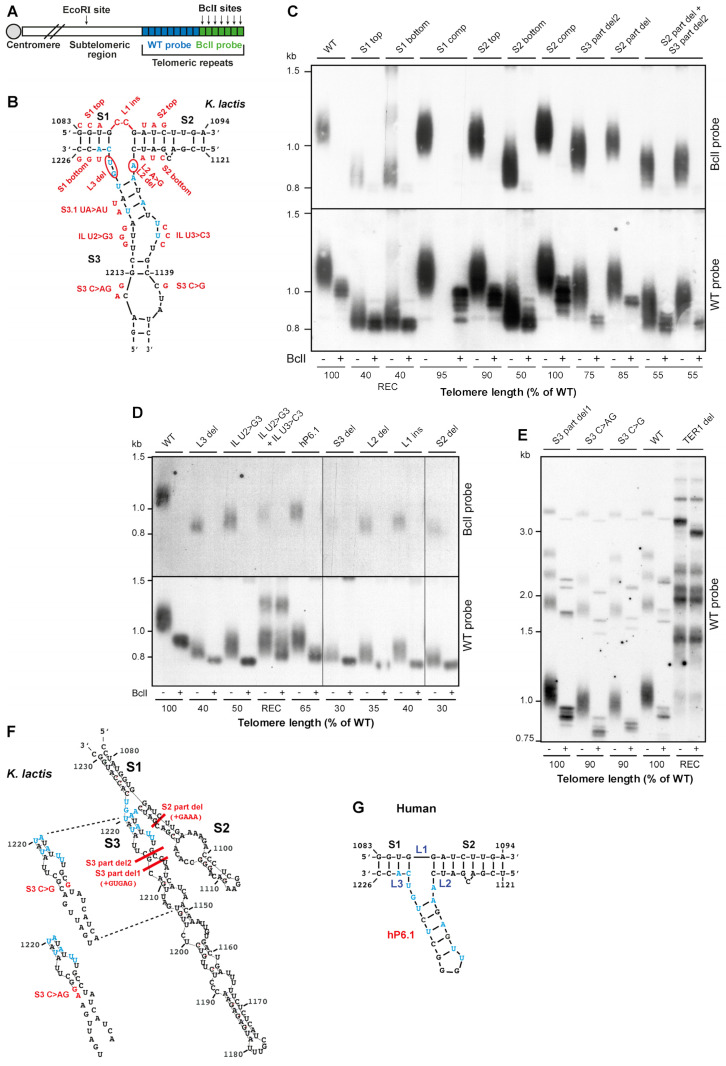
**Mutational analysis in vivo shows the importance of the precise TWJ structure for telomere maintenance.** (**A**) A schematic representation of a chromosome end of a *K. lactis* strain carrying a BclI mutation in the telomerase template, adapted from Ref. [10]. The BclI mutation is incorporated into the nascent, distal, telomeric repeats, introducing BclI restriction sites, but otherwise it does not affect telomere length. (**B**) The sequence and predicted secondary structure of the *K. lactis* TWJ is shown, with nucleotides conserved across yeast and vertebrates indicated in blue, and small substitutions and deletions introduced into the TER1 gene in vivo to study each of the structural elements in red. A list of the mutations, their positions within TER1 and their sequence is summarized in Supplementary Table S1. (**C**–**E**) Mutant *TER1-BclI* alleles were introduced into *K. lactis* cells to replace the WT *TER1* gene, as described previously [30]. Genomic DNA was prepared from the sixth passage (~90–120 generations), digested with EcoRI or EcoRI + BclI and analyzed by Southern hybridization, first with a BclI-specific telomere probe and then with a WT telomeric probe, as indicated on the right of the panels. (**F**) Small substitutions and larger deletions were introduced into stems 2 and 3 in an attempt to stabilize the TWJ structure. (**G**) Stem 3 of *kl*TWJ was replaced with human p6.1. Panel (**E**) shows the entire gel with all 12 *K. lactis* telomeres, while (**C**,**D**), for simplicity, show only the bottom portion of the gels, which includes 7 of the 12 *K. lactis* telomeres. The telomere length normalized to the WT length is indicated below the lanes. ‘REC’ indicates a pattern typical of the alternative, recombination-based mechanism for telomere maintenance.

We have previously shown that *kl*TWJ is important for telomerase action [30]. To study in more detail the predicted *kl*TWJ structure, we introduced mutations into the *kl*TWJ sequence of the *TER1* gene expressed from its endogenous promoter on a low-copy-number (CEN-ARS) plasmid. The WT *TER1* gene in *K. lactis* cells was replaced with the mutant alleles using the plasmid shuffling system previously described [30]. The mutant *TER1* genes contained an additional BclI template mutation that is incorporated into telomeres, introducing a BclI restriction site. Otherwise, the BclI mutation is phenotypically silent and can therefore be used to mark the nascent products of the investigated telomerase in vivo (Figure 2A). Telomere length and the in vivo telomerase activity were analyzed by Southern hybridization, and BclI repeats were detected by differential hybridization with a BclI-specific probe or by BclI digestion (see Materials and Methods). Three-nucleotide substitutions in either strand of stem 1 or stem 2 (Figure 2B,C; S1 and S2 top and bottom mutations) reduced telomere length to 40% (each mutation in stem 1) or 50 and 90% (bottom and top mutations in stem 2) of the WT length, while compensatory mutations in each stem (Figure 2C; S1 comp and S2 comp) restored the WT telomere length, confirming the importance of base pairings within each of stems 1 and 2. Next, we checked the importance of the predicted linkers that dictate the angles between the stems and the overall structure of *kl*TWJ (Figure 2B,D). Deleting linker 2 (L2 del) or 3 (L3 del) or inserting CC into linker 1 (L1ins) decreased telomere length to 35–40% of the normal length, indicating the importance of the precise angles between the stems. Substitutions in the U-rich internal loop reduced telomere length by 50% (IL U2 > G3) or abolished (IL U2 > G3 + IL U3 > C3) telomerase activity, as revealed by the long and heterogeneous telomeric repeats (Figure 2D) typical of the pattern observed in telomerase null mutants, maintaining their telomeres by the alternative, recombination-dependent pathway (Figure 2E, ‘REC’). Altogether, our results confirmed the importance of the precise TWJ structure for telomerase activity in vivo.

Next, we examined whether elements more distal to the junction are essential for the TWJ function. We closed an internal loop in stem 3 by two substitution mutations (S3 C > G and S3 C > AG; Figure 2F). Free energy calculations by *mfold* [36] predicted that these substitutions would stabilize the predicted *kl*TWJ structure. Each of these substitutions caused mild telomere shortening (Figure 2E), indicating that increased stability or altered structure compromised the *kl*TWJ function. Deleting the entire stem 2 or stem 3 caused severe telomere shortening to 30% of the WT telomere length (S2 del, S3 del; Figure 2D,F). Shortening stem 2 by replacing 35 apical nucleotides with a tetraloop (GAAA; S2 part del) or stem 3 by deleting 75 apical nucleotides (S3 part del2) reduced telomere length to 85% or 75% of the WT telomere length, respectively (Figure 2C,F). The combination of the two partial deletions (S2 part del + S3 part del2) had an additive effect, reducing telomere length to 55% of the WT (Figure 2C). All these manipulations compromised the ability of telomerase to maintain WT telomere length. However, deleting 72 apical nucleotides of stem 3 and replacing them with a GUGAG loop while preserving the predicted structure of the essential U-rich internal loop did not affect telomere length (S3 part del1, Figure 2E,F). We concluded that the structure and orientation of the entire stem 2 and the junction-proximal part of stem 3, including the U-rich internal loop, are important for telomerase function. Based on S3 part del1, we constructed a minimal TWJ (minTWJ) RNA construct that was 67 nt shorter than the native TWJ. minTWJ was predicted to be more stable and fold more readily than the full-length TWJ and expected to preserve the native TWJ function. This construct was used in the in vitro analysis described below.

Based on our comparative analysis, we suggested that the yeast TWJ structure is a functional homolog of the vertebrate CR4-CR5 domain, and yeast stem 3 corresponds to vertebrate P6.1, which is essential for telomerase activity [30,37]. To test whether p6.1 can substitute stem 3, we replaced yeast stem 3 with the human P6.1 stem-loop (hP6.1; Figure 2G). Indeed, human p6.1 partly suppressed the severe telomere shortening of the stem 3 deletion (S3 del; 30% of the WT length) and displayed only moderate telomere shortening—65% of the WT (Figure 2D). This result suggested that stem 3 and p6.1 are functional homologs, and thus, p6.1 can partially substitute stem 3. In conclusion, our in vivo results confirm that *kl*TWJ is a homolog of the vertebrate CR4-CR5 domain.

### 2.2. klTWJ Is Required for the Association of klEst2 with TER

**Figure 3:**
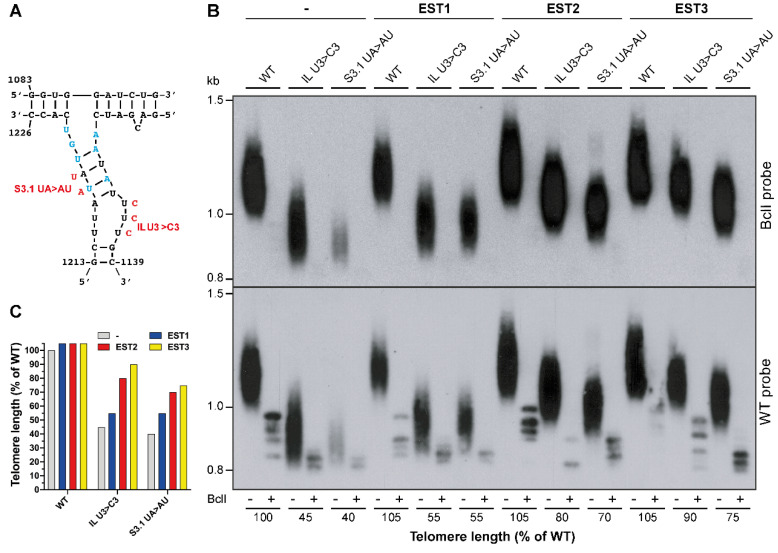
**Overexpression of *kl*Est2 and *kl*Est3 partially suppressed the short telomere phenotype of TWJ mutants.** (**A**) The indicated TWJ mutations in the helix and the U-rich internal loop of stem 3 were used for the experiment. (**B**) *K. lactis* strains carrying the WT and mutant TER1-BclI genes and a 2µ (high copy number) plasmid expressing one of the EST proteins, as indicated above the lanes, were grown for six passages and analyzed by Southern hybridization, as described in the legend in Figure 2. The telomere length normalized to the WT length was calculated and indicated below the lanes and in the bar graph shown in (**C**).

To gain insight into the TWJ function, we examined the ability of overexpressed telomerase proteins, *kl*Est1, *kl*Est2 or *kl*Est3, to suppress the short telomere phenotype of *kl*TWJ mutations. We assumed that if *kl*TWJ served as a binding site for any of these proteins, the increased concentration of interacting protein might compensate for the reduced affinity to the *kl*TWJ mutants. We examined the effect of overexpressing these proteins on *TER1* mutants carrying small substitutions in stem 3 (S3.1 UA > AU) or the U-rich internal loop (IL U3 > C3; Figure 3A). Overexpression of each of these three proteins slightly elongated the telomeres of the WT TER strains by 5% (Figure 3B,C). However, overexpression of *kl*Est2 or *kl*Est3 suppressed the short telomere phenotype of the TER mutants much more significantly, suggesting that *kl*TWJ, particularly stem 3, physically or functionally interacts with these proteins (Figure 3B,C).

**Figure 4:**
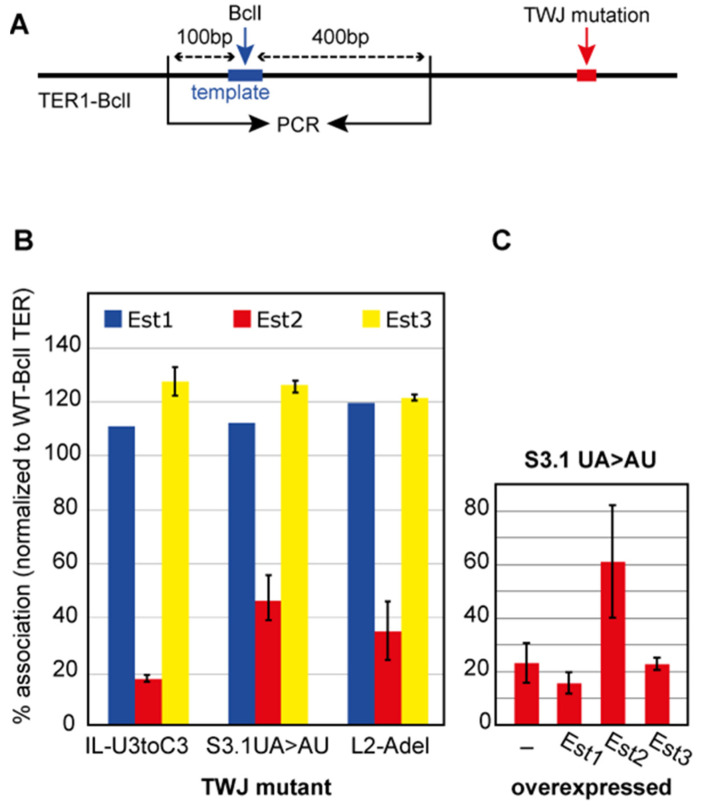
**TWJ mutations compromised the association of *kl*Est2 with TER1 in vivo.** (**A**) Whole-cell extracts were made from *K. lactis* strains expressing myc-tagged *kl*Est1, *kl*Est2 or *kl*Est3 and two TER1 genes simultaneously: TER1-WT and TER1-BclI carrying TWJ mutations. The myc-tagged protein was immunoprecipitated, total RNA was extracted, and a TER1 fragment was amplified by reverse-transcription PCR with primers flanking the template. The BclI restriction site was used to distinguish between the two amplified fragments by restriction digestion with BclI, agarose gel electrophoresis and ethidium staining. (**B**) The intensity of each of the two bands was quantified, and the fraction of TER1-BclI from the total PCR product was calculated and normalized to the IN and then to the value calculated for TER1-BclI (which was set to 100%). (**C**) A strain expressing TER1-WT, TER-BclI-S3.1 UA > AU and myc-*kl*Est2 was transformed with a high-copy-number plasmid overexpressing *kl*Est1, myc-*kl*Est2 or *kl*Est3, and the effect of the overexpressed proteins on the association of *kl*Est2 with the mutant TER1-BclI-S3.1 UA > AU was examined as in (**B**).

To examine whether *kl*TWJ mutations affect the physical interactions of these proteins with TER1, we tagged each of the *kl*Est1, *kl*Est2 and *kl*Est3 proteins with nine copies of the myc tag at their C-termini [38] by homology-directed insertion of the tag into each of these genes in their endogenous loci. We constructed strains expressing one of the tagged proteins, WT TER1 (on a CEN-ARS URA plasmid), and one of the TER1-BclI alleles (on a CEN-ARS HIS plasmid). We immunoprecipitated the tagged proteins from cell extracts using anti-myc antibody and reverse-transcribed and PCR-amplified a fragment of TER1 RNA containing the template. The BclI template mutation allowed distinguishing the relative amount of the mutant allele in the telomerase RNP complex by BclI digestion of the PCR products (Figure 4A). The association of the *kl*TWJ mutants with *kl*Est2 was reduced by 65–80%, while their association with *kl*Est1 and *kl*Est3 was not reduced but rather elevated by 10–25% as compared to WT-BclI TER (Figure 4B). These results suggest that *kl*TWJ is important for the association of *kl*Est2 with TER1. The physical association of *kl*Est1 and *kl*Est3 with TER1 was not impaired and thus did not appear to be dependent on *kl*TWJ or *kl*Est2. Their increased association may reflect a compensatory response to the compromised telomerase activity, which enhances telomerase recruitment or activation at the telomere. Finally, since *kl*Est3 overexpression partially suppressed the telomere phenotype of these mutants (Figure 3), we examined whether the effect of *kl*Est3 overexpression is mediated through stabilization of the association of *kl*Est2 with TER1. We overexpressed each of the *kl*Est1, *kl*Est2 and *kl*Est3 proteins in the background of the *kl*TWJ S3.1 UA > AU mutant and immunoprecipitated *kl*Est2. As shown in Figure 4C, only the overexpression of *kl*Est2 (but not of *kl*Est1 or *kl*Est3) increased the association of *kl*Est2 with TWJ mutants, indicating that *kl*Est3 does not contribute to *kl*Est2 binding to TER1 but rather enhances the activity of the assembled telomerase complexes.

### 2.3. The klEst2 Protein Binds In Vitro to TWJ RNA

**Figure 5:**
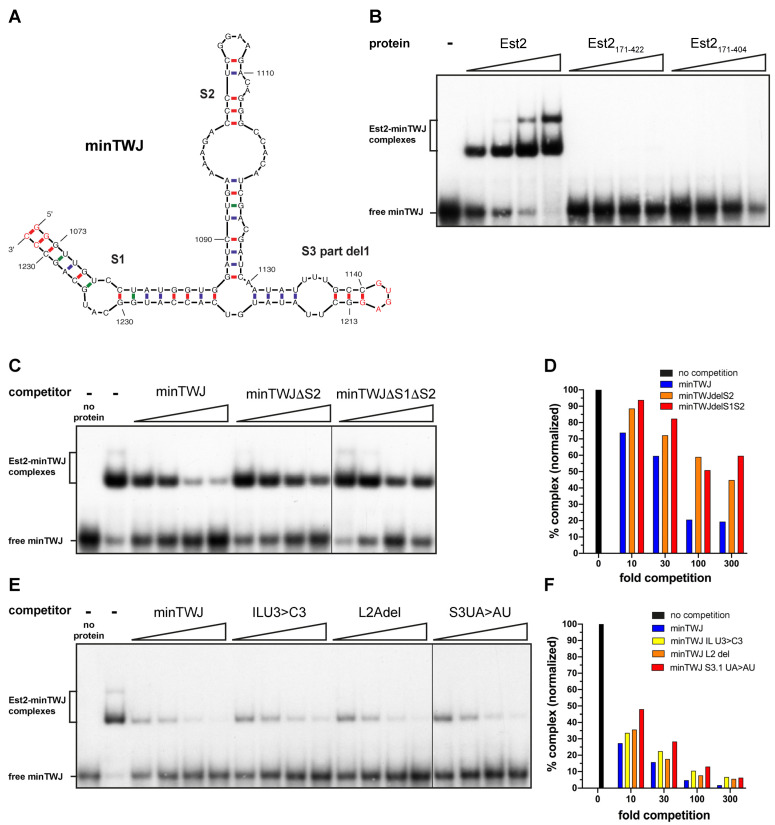
***kl*Est2 binds specifically to the TWJ structure.** (**A**) A minTWJ RNA construct, in which the apical part of stem 3 was replaced with a tetraloop, was synthesized in vitro by T7 RNA polymerase. Nucleotides not present in the WT *kl*TER1 sequence are in red. (**B**) Increasing amounts (3× each) of recombinant full-length *kl*Est2 and *kl*Est2 fragments corresponding to TRBD *kl*Est2_171-422_ and *kl*Est2_171-404_ were tested by EMSA for direct interaction with ^32^P-labeled minTWJ RNA, as described under ‘Materials and Methods’. (**C**) Unlabeled competitor RNAs (minTWJ, minTWJΔS2 or minTWJΔS1ΔS2, shown in Supplementary Figure S2) were used to compete with the labeled minTWJ at 1:10-, 30-, 100- and 300-fold molar ratios. (**E**) Unlabeled minTWJ or minTWJ competitor RNAs with the indicated small substitutions or deletion (see Figure 2B) were used to compete with the labeled minTWJ at 1:10-, 30-, 100- and 300-fold molar ratios. The protein–RNA complexes in (**C**,**E**) were quantified and normalized to the amount of complex without competitor RNA, and they are presented in the charts (**D**,**F**), respectively. ‘0′ means no minTWJ competitor was added.

Next, we tested whether *kl*Est2 binds directly to *kl*TWJ. We synthesized an RNA transcript corresponding to the partial deletion of stem 3 (S3 part del1; Figure 2F) by T7 RNA polymerase (the transcript was termed minTWJ; Figure 5A). When introduced into *kl*TER1 in vivo, this deletion of the apical part of stem 3 supported the maintenance of WT telomere length in vivo, indicating that it retained the functional structure (Figure 2E) and thus could be used to test the binding of *kl*Est2. Indeed, electrophoretic mobility shift assay (EMSA) experiments using partially purified recombinant full-length *kl*Est2 bound well to in vitro-transcribed and 5′-end-labeled minTWJ (Figure 5B, Est2-Fl). Next, we designed, expressed and purified two recombinant protein fragments: one corresponding to the *K. lactis* telomerase RNA-binding domain (*kl*Est2_171-422_), as predicted based on sequence alignment to TRBD from other species [20], and another recombinant *kl*TRBD protein fragment, *kl*Est2_171-404_, based on the limited proteolysis of *kl*Est2_171-422_, as described under Materials and Methods and Supplementary Figure S1. These *kl*TRBD polypeptides did not bind to minTWJ under the same assay conditions in which full-length *kl*Est2 bound (Figure 5B), suggesting that in *K. lactis* TRBD alone is not sufficient to form stable interactions with *kl*TWJ, and one or more other domains of the protein are required to strengthen the association. In the crystal structures of *C. albicans and C. tropicalis* telomerases, the TERT C-terminal extension (CTE) is positioned to interact with the TWJ [27].

To examine whether additional deletions in minTWJ that caused telomere shortening in vivo also compromise *kl*Est2 binding, unlabeled minTWJ constructs were used to compete with the labeled minTWJ in the EMSA reaction. Introducing additional deletions into stem 2 or both stems 1 and 2 indeed compromised the ability of the mutant minTWJ RNAs (minTWJΔS2 and minTWJΔS1ΔS2, Supplementary Figure S2) to compete with the labeled minTWJ probe for the binding of *kl*Est2 (Figure 5C–F). These results indicate that both stems 1 and 2 are important for *kl*Est2 binding, suggesting that their effect on telomerase function in vivo is due, at least in part, to the reduced affinity to *kl*Est2.

To further explore the correlation between telomere shortening in vivo, the reduced association of *kl*Est2 with TER1 in cell extracts and binding in vitro, we introduced two mutations in stem 3 (IL U3 > C3 and S3.1 UA > AU) and a deletion of the A linker between stem 2 and stem 3 (L2 del; Figure 2B) into minTWJ and transcribed the mutant minTWJ RNAs in vitro. The three mutations were shown previously to cause a rough colony phenotype and telomere shortening to ~40% of the WT length [30]. They also reduced the association of TER1 with *kl*Est2 to ~20–40% of the WT (Figure 4). Using these mutant minTWJs to compete with the labeled minTWJ revealed that these mutations only mildly affected the interaction between *kl*Est2 and minTWJ (Figure 5D,F). These results suggested that these mutations affected an additional role of TWJ in telomerase action that is independent of its role in the binding of *kl*Est2.

### 2.4. klEst2 Binds to the Template Domain

**Figure 6:**
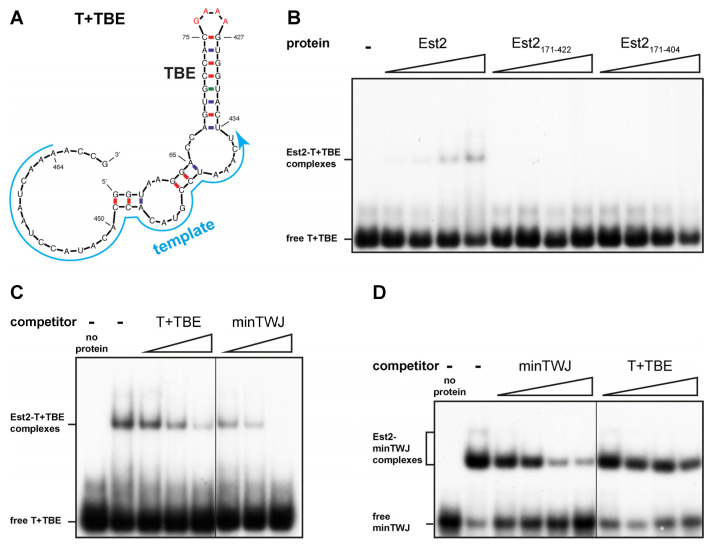
***kl*Est2 binds to the template domain.** (**A**) An RNA construct composed of the *kl*TER1 template and template boundary element (T + TBE) was synthesized in vitro by T7 RNA polymerase. The *kl*TER1 nucleotides in between the two strands of TBE were replaced with a tetraloop (GAAA; red). (**B**) Increasing amounts (3× each) of recombinant full-length *kl*Est2 and *kl*Est2 fragments corresponding to *kl*TRBD, *kl*Est2_171-422_ and *kl*Est2_171-404_ were tested by EMSA for direct interaction with ^32^P-labeled T + TBE RNA, as described under ‘Materials and Methods’. (**C**) Unlabeled T + TBE or minTWJ RNAs (Figure 5A) were used to compete with the labeled T + TBE at 1:10-, 30- and 100-fold molar ratios, as indicated above the lanes. (**D**) Unlabeled competitor minTWJ or T + TBE were used to compete with the labeled minTWJ at 1:10-, 30-, 100- and 300-fold molar ratios, as indicated above the lanes.

Next, we examined whether *kl*Est2 binds to the template domain, where it should act to elongate the telomeres, and whether the *kl*TRBD protein fragments (*kl*Est2_171-404_ and *kl*Est2_171-422_) are sufficient for this interaction. We synthesized an RNA construct composed of the template and template boundary element (T + TBE; Figure 6A) and tested it by EMSA using 1 nM of the labeled T + TBE. Protein–RNA complexes apparently formed with the full-length *kl*Est2 but not with the two *kl*TRBD constructs (Figure 6B). To investigate whether TWJ and the template domain are competing for the same binding site within *kl*Est2 or can bind simultaneously using separate binding sites, we added increasing concentrations of unlabeled T + TBE or minTWJ to the binding reaction with the labeled T + TBE at a 10-, 30- or 100-fold molar ratio of unlabeled to labeled RNA (Figure 6C). Then, we performed the reciprocal experiment, using the same unlabeled RNAs with labeled minTWJ at a 10-, 30-, 100- or 300-fold molar ratio (Figure 6D). If *kl*Est2 was binding to both RNAs simultaneously, the larger trimeric complex was expected to display different mobility and appear in a different position on the gel. However, no additional band was observed (Figure 6C,D), indicating that under these assay conditions, the interactions of *kl*Est2 with T + TBE and minTWJ were mutually exclusive, and the two RNA domains could not bind to *kl*Est2 simultaneously. minTWJ competed efficiently at all concentrations with the labeled T + TBE, while T + TBE barely competed with the labeled minTWJ (Figure 6C,D). These competition experiments suggested that the affinity of *kl*Est2 to minTWJ was higher than that to the T + TBE.

### 2.5. Structure of K. lactis TRBD

**Figure 7:**
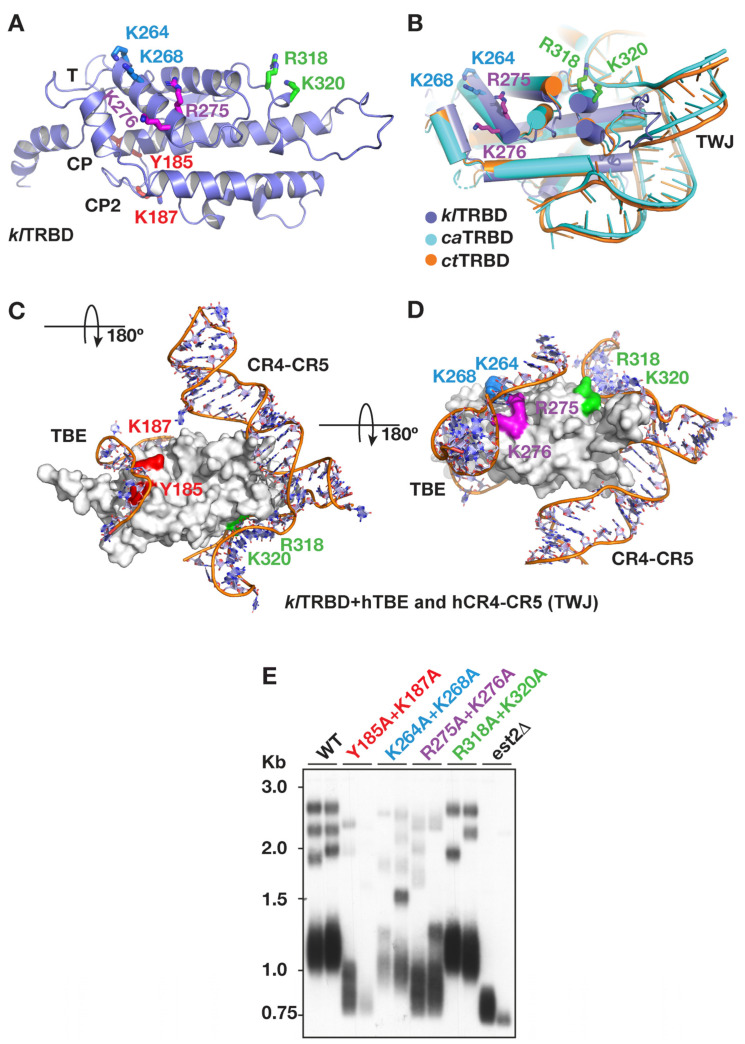
**Crystal structure and mutagenesis of surface amino acid residues of *kl*TRBD.** (**A**) Crystal structure of *kl*TRBD (PDB ID: 7SBE) in cartoon representation. Residues mutated in this study are shown as colored sticks and labeled. Motifs T, CP and CP2 are indicated. (**B**) The *kl*TRBD structure docked on the solved crystal structures of *C. albicans* (ca) and *C. tropicalis* (ct) TERT proteins assembled with their respective TWJ RNAs [27]. The structural overlay was made in *Pymol* [39]. (**C**,**D**) Surface representation of *kl*TRBD structure with human telomerase RNA from (25) docked using a structural overlay in *Pymol* [39]. The structure in (**C**) is rotated 180° with respect to (**A**,**B**,**D**). Residues mutated in this study (to alanine), Y185 + K187, K264 + K268, R275 + K276 and R318A + K320A, are highlighted in matching colors. (**E**) WT and mutant *klEst2* genes were introduced into a *K. lactis est2Δ* strain on a CEN-ARS plasmid, and two clones from each strain were passaged. Genomic DNA was prepared from the sixth passage, digested with EcoRI and analyzed by Southern hybridization with a WT telomeric probe.

To identify a stable fragment of *kl*Est2 corresponding to *kl*TRBD that would readily crystalize, we applied limited proteolysis coupled with mass spectrometry to the partially purified full-length *kl*Est2 protein, as described under Materials and Methods and shown in Supplementary Figure S1. Based on this analysis, we overexpressed it in *E. coli* and purified the *kl*TRBD polypeptide *kl*Est2_171-404_ to apparent homogeneity. We subsequently crystallized the protein and solved the structure to 2.65 Å resolution (Table 1 and Figure 7A). Despite the low sequence identity (<21%) within the N-terminal domains (NTE and TRBD) of *kl*TERT and other budding yeasts, the *kl*TRBD structure revealed a remarkable structural homology to previously solved TRBD structures from human (RSMD: 1.8 Å), *Tetrahymena* (RSMD: 2.2 Å), *Tribolium* (RSMD: 2.3 Å)*, Fugu rubripes* (RSMD: 1.7 Å), *C. albicans* (RSMD: 1.9 Å) and *C. tropicalis* (RSMD: 1.9 Å) [17,20,21,25,27], with an average RMSD (for all structures) of ~2.1 Å (Figure 7B–D). It is an elongated helical bundle that includes the conserved CP2/TFLY, CP, T and CR4-CR5 binding motifs. The CP2/TFLY, CP and T motifs are known to form a pocket at the interface of the TRBD and finger domains, which interact with the template boundary element (TBE) of telomerase RNA (Figure 7 and [20,21]). It is worth noting that based on several TRBD structures determined, the CP2 motif is highly flexible in the absence of the TBE, adopting a variety of secondary structures and locations on the surface of TRBD.

### 2.6. In Vivo Interactions of K. lactis TRBD with klTER1

The solved crystal structure of *kl*TRBD revealed several surface patches of positively charged or polar residues that might be involved in RNA binding (Figure 7A). Docking human telomerase RNA with the *kl*TRBD structures using a structural overlay, based on the solved human telomerase RNA structure [25], suggested that lysine residues 187, 264, 268 and 276, arginine 275 and tyrosine 185 interact with the template boundary element (TBE), while arginine 318 and lysine 320 interact with the TWJ-homologous domain CR4-CR5 (Figure 7B,D). To test these predictions, we made alanine substitutions of these residues in pairs (Figure 7C) and introduced the mutant *klest2* gene, under its endogenous promoter in a CEN-ARS (low copy number) plasmid, into a *K. lactis est2Δ* strain. Two clones from each strain were grown to the sixth passage, and telomere length was analyzed by Southern blot (Figure 7E). The double mutants Y185A + K187A and R275A + K276A displayed severely short telomeres, and K264A + K268A caused milder but still significant telomere shortening, indicating that at least one residue from each pair is important for RNA binding, presumably in the template domain. On the other hand, the mutations R318A + K320A, which were predicted to affect binding to *kl*TWJ, did not affect telomere length, suggesting that these residues are not essential or are redundant for the interaction of *kl*Est2 with *kl*TER1.

## 3. Discussion

We previously identified a TWJ structure that is essential for *K. lactis* telomerase function and is conserved in all fungal TERs [6,30,31,32]. Furthermore, the yeast TWJ displayed conserved features with the vertebrate CR4-CR5 domain, suggesting a common function between these TER domains [30]. Vertebrate CR4-CR5 and ciliate stem IV were shown to bind TERT [21,33,34,35] and have been suggested to constitute functional homologs [40,41]. In addition to the previously reported triplex-containing pseudoknot [10,12,13], the TWJ demonstrates that, while TERs are highly divergent in sequence and length, they do share conserved elements providing conserved telomerase functions. These elements are embedded in non-conserved RNA fragments, as suggested by the ‘beads on a string’ model [42]. Interestingly, while the *S. cerevisiae* TWJ (*sc*TWJ) is conserved in all fungal TERs examined [30], it was found to be dispensable for telomerase activity [29,42]. The loss of the essential function of *sc*TWJ correlates with the reduced processivity of *S. cerevisiae* telomerase along the template and the redundant telomeric sequences incorporated, possibly indicating a role for the TWJ in telomerase processivity. This hypothesis is yet to be examined. Here, we further examined the *kl*TWJ structure and found that the proximal parts of stems 1 and 3 (including the U-rich internal loop in stem 3), all of stem 2 and the precise linkers between the three stems are all important for telomerase function in vivo (Figure 2). The precise TWJ structure is also important for the stable association of TER1 with *kl*Est2, since mutations that compromise the TWJ structure disrupt the *kl*Est2–TER1 association in immunoprecipitation assays (Figure 4). Furthermore, overexpression of *kl*Est2 partially suppressed the short telomere phenotype and increased the *kl*Est2 association with the TWJ mutants (Figure 2 and Figure 3), suggesting that the TWJ mutations reduced the affinity of the interaction between *kl*Est2 and TER1. In contrast, TWJ mutations did not reduce the association of *kl*Est1 or *kl*Est3 with TER1, and while overexpression of *kl*Est3 partially suppressed the short telomere phenotype, it did not increase the association of *kl*Est2 with the TWJ mutants. These results suggest that *kl*Est3 overexpression did not restore complex assembly but rather suppressed the short telomeres of TWJ mutants by enhancing the recruitment or activity of the assembled telomerase to the telomeres.

Full-length *kl*Est2 bound specifically to *kl*TWJ at 1 nM RNA concentration in vitro, as shown by EMSA experiments (Figure 5). This interaction requires all of stem 2, consistent with what has been observed in the *Medaka* TRBD-CR4-CR5 structure and Candida TERT-TWJ structures [21,27]. However, the *kl*TRBD fragments (*kl*Est2_171-422_ and *kl*Est2_171-404_) did not bind under the same assay conditions. The measured *Kd* values for the interactions of recombinant TRBD proteins from *Medaka* and *Schizosaccharomyces pombe* with CR4-CR5 and TWJ are 0.76 and 0.57µM, respectively [21]. Assuming that *kl*TRBD has a similar *Kd* for binding to *kl*TWJ, it is not expected to bind at RNA concentrations almost 3 orders of magnitude lower. The ability of the full-length *kl*Est2 to bind RNA at such a low concentration indicates that additional domains of *kl*Est2 contribute to the binding. Indeed, current structural telomerase data show extensive interactions between the TRBD and CTE domains of TERT and TER CR4-CR5 in vertebrates, stem-loop IV in ciliates and the fungal TWJ [4,21,24,26,27]. More specifically, TRBD makes extensive contacts with the P6 and P6.1 stem-loops of CR4-CR5 (corresponding to stems 2 and 3, respectively, in yeast), while the tip of stem-loop P6.1 reaches across and coordinates the CTE domain where the FVYL pocket is located [27]. Interestingly, yeast stem 3 is significantly longer (90 nucleotides) than vertebrate P6.1 (13 nucleotides), potentially making more contacts with the CTE than P6.1. Therefore, P6.1 could not fully substitute for stem 3 (Figure 2D,G). To further explore the interactions of *kl*TRBD with TER, we used a structural overlay of human, *Medaka* and *K. lactis* TRBD structures to identify residues of *kl*TRBD that are within coordinating distance of stem-loop P6.1 of CR4-CR5 (yeast stem 3). Interestingly, alanine substitution of such residues, R318 and K320, did not have an apparent effect on telomere length in vivo, suggesting that the interaction of *kl*Est2 with stem 3 is not mediated by TRBD as in vertebrates but rather by CTE. This is not surprising considering the significant differences in size between P6.1 and *K. lactis* stem 3. It is possible that the two stem-loops adopt different configurations in relation to the rest of TER, which place the *kl*TRBD residues R318 and K320 away from the TWJ. On the other hand, alanine substitution of Y185 + K187 or R275 + K276 caused severe telomere shortening (Figure 7C). Y185 and K187 are at the interface of TRBD and the RT domains and form part of the CP2 motif, which, in human and *Tetrahymena* telomerase, engage the template boundary element (Figure 7C). R275 and K276 form part of the helix located adjacent to CP and interact with the TBE loop that connects it to the pseudoknot in human telomerase (Figure 7C,D). Therefore, it is possible that these interactions may not necessarily be required for the stable association of *kl*Est2 and TER but rather for the catalytic activity of the telomerase complex, and therefore, they are more sensitive to mutagenesis. Altogether, our results reveal specific interactions of *kl*Est2 and TER that are essential for telomerase activity and demonstrate that co-evolution of telomerase RNA and reverse-transcriptase protein largely conserved these interactions across ciliates, vertebrates and yeast but also allowed some differences to evolve.

## 4. Materials and Methods

### 4.1. Yeast Strains TER1 and klEst2 Mutagenesis

WT or mutant *TER1* genes were introduced into *K. lactis* strain yJR27 (*ter1∆ ura3-1 his2-2 trp1*) on a CEN-ARS HIS plasmid, replacing the WT *TER1* gene on a CEN-ARS URA plasmid, by plasmid shuffling as described in [43]. The investigated TER alleles contained an additional BclI template mutation that is incorporated into telomeres, introducing a BclI restriction site (Figure 2A). Otherwise, the BclI mutation is phenotypically silent and can therefore be used to mark the nascent products of the investigated telomerase in vivo [43]. The *klEst2* (WT and mutants) alleles were cloned into a CEN-ARS URA plasmid and introduced into *K. lactis* strain yKU1 *(est2∆ ura3-1 his2-2 trp1*). All yeast strains were grown at 30 °C.

### 4.2. Tagging klEst1, klEst2 and klEst3 Proteins with Myc Tag

The *K. lactis EST1, EST2* and *EST3* genes were C-terminal tagged in their endogenous genomic locations with 9 x myc and a G8 spacer by homologous recombination [39]. The cassette with the 9× myc tag and a Nourseothricin (NAT) selection marker was inserted into the parental yJR27 strain at the C-terminus of the indicated proteins to generate the following strains: yMK11 (*EST1-myc::NAT*), yMK12 (*EST2-myc::NAT*) and yMK13 (*Est3-myc::NAT*). All strains were confirmed by PCR and sequencing, and their telomere length was found to be undistinguishable from the WT length in Southern analysis.

### 4.3. Overexpression of Telomerase Proteins In Vivo

The *K. lactis EST1*, *EST2* and *EST3* genes were cloned into a *K. lactis* 2 μm-based high-copy-number plasmid (pKDU7) with a *URA3* selection marker, as described in [44]. These plasmids, along with the pKDU7 parental plasmid as a control, were introduced into *K. lactis* cells containing a WT or mutant *ter1* gene on a CEN-ARS plasmid. The overexpression of these genes was verified by Northern blot analysis [44]. For RNA pull-down experiments using *kl*Est2-myc while overexpressing *kl*Est1 or *kl*Est3, the yYB01 strain (*EST2-myc::NAT, his2, ura3* and *TER1*) was generated by mating and sporulation of yMK12 with the TER WT strain 7B520. yYB01 was transformed with pKDU7-based plasmids encoding *kl*Est1, *kl*Est3 or *kl*Est2-myc.

### 4.4. Southern Analysis of Telomeric Fragments

Genomic DNA was prepared from the sixth passage (90–120 generations), digested with EcoRI or EcoRI + BclI, electrophoresed on a 1% agarose gel, vacuum blotted onto a membrane and hybridized to a telomeric probe as described in [12].

### 4.5. Co-Immunoprecipitation

*K. lactis* strains were constructed to express myc-tagged *kl*Est1, *kl*Est2 or *kl*Est3 from their endogenous chromosomal loci, WT TER1 from a CEN-ARS URA plasmid and TER1-BclI (WT or TWJ mutants) from a CEN-ARS HIS plasmid. Total RNA was prepared from cell extracts before and after immunoprecipitation with anti-myc antibody, reverse-transcribed and PCR-amplified with TER1-specific primers flanking the template to produce a 500 bp fragment with the silent BclI template mutation about 100 bp from the end. PCR products were digested with BclI and electrophoresed in an agarose gel. The intensity of the bands was used to calculate the percentage of the BclI-digested TER1 from the total TER1 transcript. Note that the *kl*Est1 pull-down was performed only once.

### 4.6. klTRBD Subcloning

The *kl*TRBD domain, *kl*Est2 residues 171-422 (*kl*Est2_171-422_), was first chosen based on protein sequence alignment to available TRBD sequences of other species. This *kl*TRBD polypeptide was subjected to limited proteolysis to define a smaller *kl*TRBD fragment (*kl*Est2_171-404_), as described below. The sequences encoding for *kl*Est2_171-422_ and *kl*Est2_171-404_ were subcloned into the pET-28b expression vector containing a Tobacco Etch Virus (TEV) protease-cleavable His-Small Ubiquitin-like Modifier (SUMO) tag at its N-terminus using ligation-independent cloning (LIC). The ligation products were transformed into DH10β cells (Invitrogen, Waltham, MA, USA), and the cells were plated and grown at 37° C overnight. Random colonies were chosen from the plates to be grown overnight and harvested by centrifugation, and the plasmid was isolated using the QIAprep Miniprep kit from QIAGEN (Valencia, CA, USA). Clones containing the correct insert were confirmed through restriction enzyme digestion and DNA sequencing at the Wistar Sequencing Facility.

### 4.7. Protein Expression and Purification

Overexpression and solubility tests of *kl*Est2 and *kl*TRBD (*kl*Est2_171-404_ and *kl*Est2_171-422_) proteins using a wide range of *E. coli* cell lines identified the RIPL competent cell line (from Stratagene, Agilent Technologies, Santa Clara, CA, USA) as ideal for obtaining sufficient quantities of the protein for the proposed studies. We overexpressed the protein (in RIPL cells) for 6 h at 20 °C. The cells were harvested by centrifugation, and the pellet was resuspended in 25 mM Tris, 1 M KCl, 1 M Urea, 5% Glycerol and 25 mM Imidazole, frozen and stored at −80° C. For protein purification, the cells were thawed at room temperature, sonicated to lyse the cells and centrifuged to remove insoluble material.

The first purification step involved the Superflow Ni-Nitrilotriacetic acid (Ni.NTA; Qiagen) column. The soluble lysate fraction was loaded onto Ni.NTA resin equilibrated with 5% buffer B (25 mM Tris, 1 M KCl, 1 M Urea, 5% Glycerol and 25 mM Imidazole). The sample loaded on the column was washed thoroughly with 5% buffer B and then buffer C (25 mM Tris, 0.5 M KCl and 5% Glycerol) to remove any contaminant proteins, and the protein of interest was eluted from the column using a 25 to 500 mM Imidazole gradient. The fractions containing the peak(s) representing the protein of interest were run on a 12% SDS-PAGE gel to determine the protein expression levels and protein purity. The fractions containing the protein of interest were combined and concentrated to a smaller volume using a 10 K cut-off Amicon filter and incubated with TEV protease overnight at 4 °C to cleave the fusion (His-SUMO) tag. The protein sample was then further purified over tandem Poros-HS and HQ columns (Perspective Biosystems, Framingham, MA, USA) to remove any protein contaminants carried over from the Ni.NTA step and the cleaved tag. The final step of purification utilized a size-exclusion Superdex 200 (GE Healthcare) column to remove soluble protein aggregates.

### 4.8. Limited Proteolysis

The *kl*Est2_171-404_ fragment was generated by V8 digestion as follows: The *kl*TRBD_171-422_ protein fragment was made in buffer containing 25 mM Tris, 5% glycerol, 500 mM KCl and 1 mM TCEP, pH 7.5. Then, 5 µL of 5 mg/mL *kl*TRBD_171-422_ was incubated at room temperature with V8 or chymotrypsin protease (Sigma, Burlington, MA, USA) at a 1:100 ratio (V8:klTRBD) for 1, 5, 30 and 60 min. The reaction was stopped by adding Laemmli buffer, and the products were run on SDS-PAGE (Supplementary Figure S1). Using mass spectrometry analysis, the band highlighted with a red box was identified to contain a protein fragment corresponding to *kl*TRBD_171-404_, which was then subcloned and overexpressed for subsequent studies.

### 4.9. Electrophoretic Mobility Shift Assay

Recombinant proteins were expressed in *E. coli* and partially purified (*kl*Est2) or purified to apparent homogeneity (*kl*Est2_171-404_ and *kl*Est2_171-422_) as described above. RNA constructs were in vitro transcribed with T7 RNA polymerase using a DNA template that was amplified from the mutant TER1 genes in the plasmids used for in vivo mutagenesis while adding the promoter sequence for T7 RNA polymerase at the 5′ end. Transcripts were gel-purified, 5′-dephosphorylated by recombinant Shrimp Alkaline Phosphatase (NEB Inc., Ipswich, MA, USA) and end-labeled using T4 polynucleotide kinase (NEB) and [γ-^32^P] ATP. Binding reactions contained 0.5 mg/mL BSA, 1.7 ng/µL yeast tRNA, 0.1 % triton, 25 mM Tris-HCl pH 7.5, 50 mM KCl, 10% Glycerol, 1 mM DTT, 1 mM MgCl_2_ and 1 nM RNA probe. Reactions were incubated at 65 °C for 2 min and chilled on ice, then *kl*Est2 protein was added, and the reactions were incubated again on ice for 15 min and then at room temperature for 10 min before loading onto a 4.5% polyacrylamide gel (1:40 bisacrylamide:acrylamide) in 0.5× TBE buffer. Samples were run at 7.5 V/h and 6 °C for 2 h. After running, the gel was dried and exposed to Typhoon FLA 9500 PhosphorImager (GE Healthcare Inc., Boston, MA, USA).

### 4.10. Protein Crystallization

The purified *kl*TRBD protein (*kl*Est2_171-404_) was concentrated to approximately 10–15 mg/mL and dialyzed in buffer containing 10 mM Tris, 100 mM KCl and 1 mM TCEP, pH 7.5, for 3 h to remove excess salt and glycerol for crystallographic studies. For *kl*TRBD crystallization alone, crystallization trials were performed using the sitting drop vapor diffusion method and a range of sparse matrix conditions commercially available from Hampton Research. Crystal trials produced a crystal hit in the PEG ION screen consisting of 20% PEG3350 and 200 mM Na.citrate. For crystal optimization, we tested different citrate salts and the concentration of the precipitant, PEG3350. More specifically, the crystallization trials consisted of either 200 mM ammonium citrate or lithium citrate and a concentration gradient of PEG3350: 8%, 10%, 12%, 14% and 16%. Crystals appeared within 3–5 days of setting the crystal trays and grew to maximum size (100 × 30 × 500 microns) within 2 weeks. We determined through the crystallization trials that the best conditions for quality crystals in terms of size and morphology are 200 mM Ammonium citrate with 10–13% PEG 3350. The remainder of the protein was used to set crystal trays with these conditions so that a pool of crystals could be isolated for X-ray diffraction studies.

### 4.11. Data Collection and Structure Determination

X-ray diffraction data were collected at the National Synchrotron Light Source with beamline X25. The crystals diffracted to 2.65 Å resolution and belong to the monoclinic space group C2 with two molecules in the asymmetric unit. The data were processed using the software XDS [45], and the structure was determined by molecular replacement using the TRBD structure of *Tetrahymena thermophila* (*tt*TRBD:PDB ID:2R4G) [20] previously published by our lab. The structure was modeled using Coot, and the model was refined using REFMAC [46] in Phenix [47]. The coordinates have been deposited in the RCSB database, and the PDB ID is 7SBE. Data collection and refinement statistics are shown in Table 1.

## Figures and Tables

**Table 1:** Data collection and refinement statistics.

*kl*TRBD	Native
**Data collection**	
Space group	C2
Cell dimensions	
*a*, *b*, *c* (Å), *β* (°)	140.37, 85.00, 48.42, 106.109
Resolution (Å)	20–2.65 (2.72–2.65) *
CC(1/2)	99.7 (58.0)
*I*/s*I*	9.9 (0.9)
Completeness (%)	99.3 (99.7)
Redundancy	5.4 (5.5)
**Refinement**	
Resolution (Å)	20-2.65
No. of reflections	15,832
*R*_work_/*R*_free_	23.6/28.4
No. atoms	
Protein	3796
Ligand/ion	0
Water	9
Mean B value	49
R.m.s. deviations	
Bond lengths (Å)	0.004
Bond angles (°)	0.724
Ramachandran Plot	
Favored	93.14
Allowed	6.86
Outliers	0.0

* Values in parentheses are for highest-resolution shell.

## Data Availability

The crystal structure coordinates of *kl*TRBD have been deposited in the RCSB database and the PDB ID is 7SBE.

## Supplementary Materials

The following supporting information can be downloaded at: https://www.mdpi.com/article/10.3390/ijms231810757/s1.
