# Supplementary material for: Functional Interactions of Kluyveromyces lactis Telomerase Reverse Transcriptase with the Three-Way Junction and the Template Domains of Telomerase RNA
Source: Int J Mol Sci. 2022 Sep 15;23(18):10757. doi: 10.3390/ijms231810757 (PMC9504884; doi:10.3390/ijms231810757)
Supplement: Supplementary file 1 [file ijms-23-10757-s001.zip › ijms-1903604-supplementary.pdf]

Functional interactions of the yeast telomerase reverse transcriptase with the three-way junction and the template domains of telomerase RNA

Wasif Al-Shareef, Yogev Brown, Chris Bryan, Elena Shuvaeva, Shhadeh Bsoul, Raanan Greenman, Majdi M. Kabaha, Nikolai B. Ulyanov, Emmanuel Skordalakes and Yehuda Tzfati

Supplementary Materials

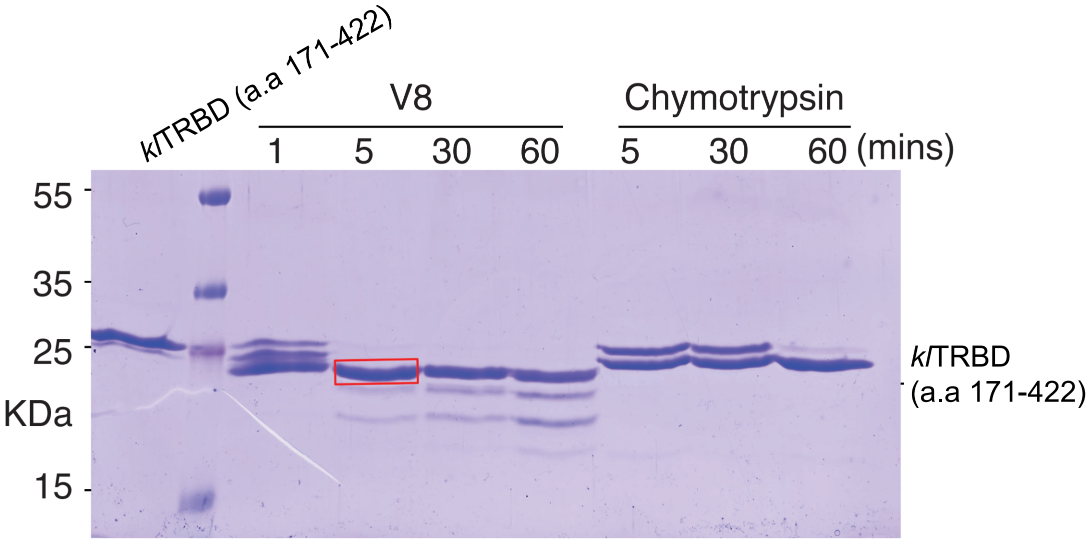

Figure S1. *kl*TRBD proteolysis. The recombinant *kl*TRBD protein fragment defined by sequence alignment, *kl*TRBD (171-422), was incubated with V8 or chymotrypsin protease at 1:100 ratio (V8 : *kl*TRBD), for 1, 5, 30 and 60mins. The reaction was stopped by incubating the sample with SDS loading buffer and run on an SDS PAGE gel. The band marked by a red box was analyzed be mass spectrometry and found to consist of residues 171-404.

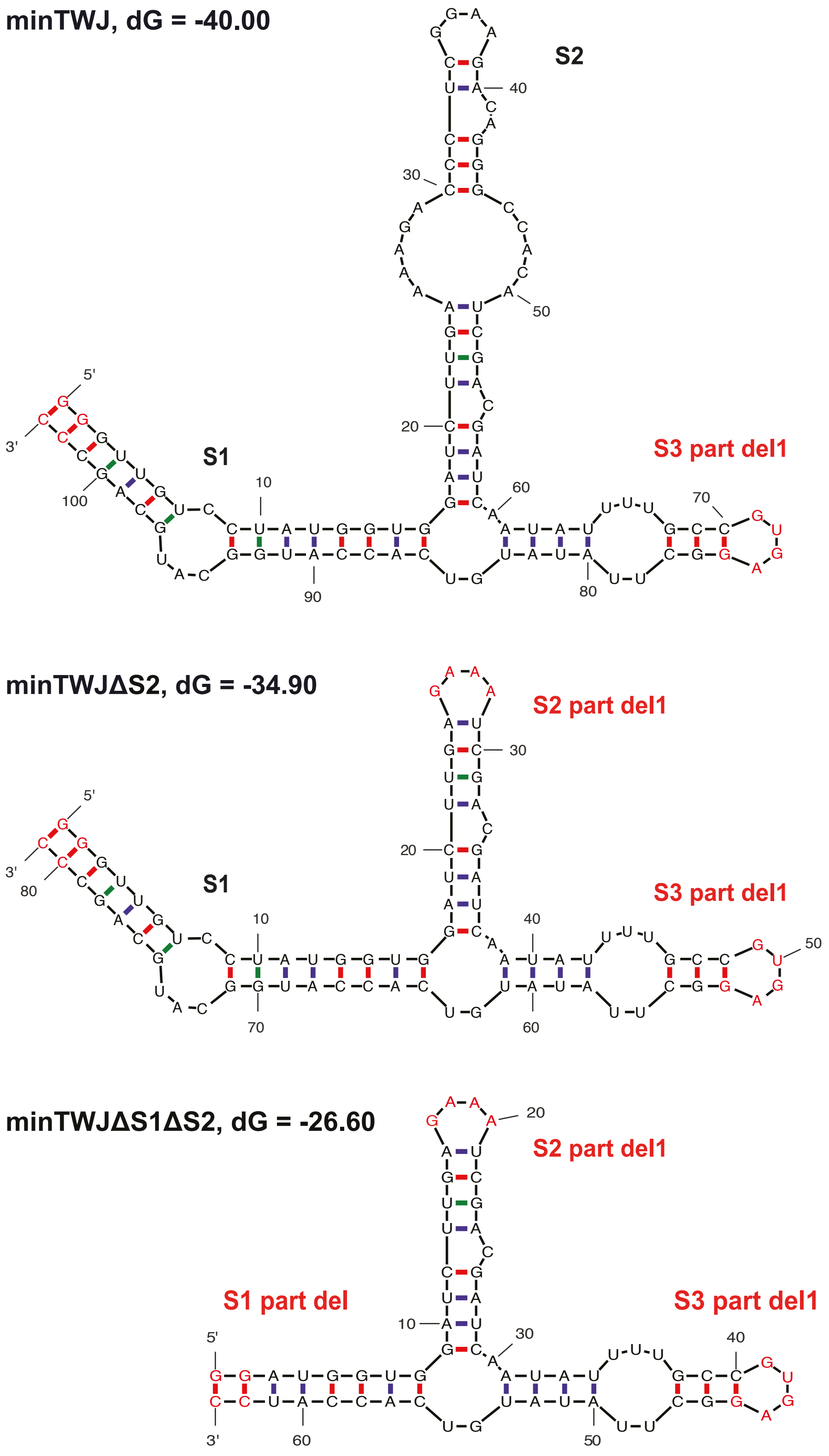

Figure S2. *In vitro* transcribed minTWJ RNA constructs. Structures and minimal energies (dG) of minTWJ, minTWJΔS2 and minTWJΔS1ΔS2 were predicted by *mfold*. Indicated in red are nucleotides substituted from the native TWJ sequence.

Table S1. TER1 mutations summary.

Name	TWJ Element	Position within TER1	WT sequence	Inserted sequence	Telomere length (% of WT)	Description
S1 top	Stem 1	1083-6	GGU	CCA	40	Transversion substitution in CS5a and stem 1
S1 bottom	Stem 1	1224-6	ACC	UGG	40, REC	Transversion substitution in CS6 and stem 1
S1 comp	Stem 1	1083-6<br>1224-6	GGU<br>ACC	CCA<br>UGG	95	S1 top+S1 bottom
S2 top	Stem 2	1088-90	AUC	UAG	90	Transversion substitution in CS5a and stem 2
S2 bottom	Stem 2	1126-8	GUA	CUA	50	Transversion substitution in CS5 and stem 2
S2 comp	Stem 2	1088-90<br>1126-8	AUC<br>GUA	UAG<br>CUA	100	S2 top+S2 bottom
S2 part del	Stem 2	1091-125	35 nt	Del +GAAA	85	Partial deletion of stem 2 + tetraloop
S2 del	Stem 2	1087-129	43	Del	30	Deletion of stem 2
S3 part del1	Stem 3	1141-212	72 nt	Del + GUGAG	100	Partial deletion of stem 3 + tetraloop
S3 part del2	Stem 3	1139-213	75 nt	Del	75	Partial deletion of stem 3
S2 part del + S3 part del2	Stem 2<br>Stem 3	1091-125<br>1139-213	35 nt<br>75 nt	Del +GAAA<br>Del	55	Partial deletions in stem 2 and stem 3
IL U2>G3	Stem 3	1215-7	AUU	GGG	50	Substitution in the internal loop
IL U2>G3 +IL U3>C3	Stem 3	1215-7<br>1135-7	AUU<br>UUU	GGG<br>CCC	REC	Closure of the internal loop with three G-C bp
hP6.1	Stem 3	1132-219	88 nt	GAGUUGG GCUC	65	Replacement all stem 3 with the human P6.1
S3 del	Stem 3	1130-222	93	Del	30	Deletion of stem 2
S3 C>AG	Stem 3	1212	C	AG	90	Transversion substitution in stem 3 to pair the internal loop
S3 C>G	Stem 3	1140	C	G	90	Transversion substitution in stem 3 to pair the internal loop
L1 ins	Stem 1 - Stem 2 Junction	1186-	-	CC	40	Insertion of linker 1
L2 del	Stem 2 - Stem 3 Junction	1130	A	Del	35	Deletion of linker 2
L3 del	Stem 1 - Stem 3 Junction	1221-2	GU	Del	40	Deletion of linker 3

REC, alternative recombination pathway for telomere maintenance

Del, deletion
